# The effect of proteoglycans inhibited by RNA interference on metastatic characters of human salivary adenoid cystic carcinoma

**DOI:** 10.1186/1471-2407-9-456

**Published:** 2009-12-21

**Authors:** Hong Shi, Jie Wang, Fusheng Dong, Xu Wang, Hexiang Li, Yali Hou

**Affiliations:** 1 Department of Oral Pathology, Key Laboratory of Stomatology, College of Stomatology, Hebei Medical University, Hebei Province, No. 383 Zhongshan East Road, Shijiazhuang, PR China; 2 Department of Oral and Maxillofacial surgery, College of Stomatology, Hebei Medical University, Hebei Province, No. 383 Zhongshan East Road, Shijiazhuang, PR China

## Abstract

**Background:**

Salivary adenoid cystic carcinoma (SACC) is one of the most common malignancies of salivary gland. Recurrence or/and early metastasis is its biological properties. In SACC, neoplastic myoepithelial cells secrete proteoglycans unconventionally full of the cribriform or tubular and glandular structures of SACC. Literatures have demonstrated that extracellular matrix provided an essential microenvironment for the biological behavior of SACC. However, there is rare study of the effect of proteoglycans on the potential metastasis of SACC.

In this study, human xylosyltransferase-I (XTLY-I) gene, which catalyzes the rate-limited step of proteoglycans biosynthesis, was knocked down by RNA interference (RNAi) to inhibit the proteoglycans biosynthesis in SACC cell line with high tendency of lung metastasis (SACC-M). The impact of down-regulated proteoglycans on the metastasis characters of SACC-M cells was analyzed and discussed. This research could provide a new idea for the clinical treatment of SACC.

**Methods:**

The eukaryotic expression vector of short hairpin RNA (shRNA) targeting XTLY-I gene was constructed and transfected into SACC-M cells. A stably transfectant cell line named SACC-M-WJ4 was isolated. The XTLY-I expression was measured by real-time PCR and Western blot; the reduction of proteoglycans was measured. The invasion and metastasis of SACC-M-WJ4 cells were detected; the effect of down-regulated proteoglycans on the potential lung metastasis of nude mice was observed, respectively.

**Results:**

The shRNA plasmid targeting XTLY-I gene showed powerful efficiency of RNAi. The mRNA level of target gene decreased by 86.81%, the protein level was decreased by 80.10%, respectively. The silence of XTLY-I gene resulted in the reduction of proteoglycans significantly in SACC-M-WJ4 cells. The inhibitory rate of proteoglycans was 58.17% (24 h), 66.06% (48 h), 57.91% (72 h), 59.36% (96 h), and 55.65% (120 h), respectively. The reduction of proteoglycans suppressed the adhesion, invasion and metastasis properties of SACC-M cells, and decreased the lung metastasis of SACC-M cells markedly either.

**Conclusion:**

The data suggested that the silence of XTLY-I gene in SACC-M cells could suppress proteoglycans biosynthesis and secretion significantly. The reduction of proteoglycans inhibited cell adhesion, invasion and metastasis of SACC-M cells. There is a close relationship between proteoglycans and the biological behavior of SACC.

## Background

Proteoglycans are important macromolecules, which show the largest and most complex molecular structures in human body. Proteoglycans are also the major components of the extracellular matrix and consist of core protein and glocosaminoglycans (GAGs) chains which attached to the core protein. Proteoglycans are increasingly implicated as important regulators in many biological processes, such as extracellular matrix deposition, cytoplasmic membrane signal transfer, cell differentiation, adhesion and migration, normal and tumor cell proliferation, etc [1-3]. With the development of studies in proteoglycans, more and more researchers have paid attention to the function of proteoglycans in tumorigenesis and biological behavior of tumors.

Proteoglycans mainly composed of core protein and GAGs chains are polyanionic molecules located in the extracellular matrix or the cell surface and serve a wide range of functions. Proteoglycans mediate diverse cellular processes through interaction with a variety of cytokines and protein ligands and there are many cell factors in themselves too. The GAGs chains attached to the core protein are involved in most of these functions by holding different cytokines and ligands. Thus, the biological feature and function of proteoglycans are intimately related to the biosynthesis of GAGs chains [3-5]. The sulfated GAGs which are the major proteoglycans components, such as chondroitin sulfate, heparan sulfate, heparin, and dermatan sulfate, etc. are bound to the proteoglycans core protein by a common xylose-galactose-galactose binding region: a tetrasaccharid core (GlcAβ1-3Galβ1-3Galβ1-4Xylβ1-O-Ser). Xylosyltransferase-I (XTLY-I) is the chain-initiating enzyme in the biosynthesis of this tetrasaccharid core of glycosaminoglycan-containing. This enzyme catalyzes the transfer of xylose from UDP-xylose to selected serine residues in the proteoglycans core protein, which is the initial and rate-limited step in the proteoglycans biosynthesis of human. XTLY-I is a key to the biosynthesis of this tetrasaccharid core, which is shared by most proteoglycans, so it has been thought that XTLY-I is a regulatory factor in proteoglycans biosynthesis [6,7]. As XTLY-I is the initial enzyme in the biosynthesis of the glycosaminoglycan linkage region and secreted from the Golgi compartment into the extracellular space together with proteoglycans to a great extent, it had been determined that the activity of XTLY-I is a crucial and diagnostic biochemical marker of an altered proteoglycans biosynthesis in human body. The effect of XTLY-I on human health and diseases has become a new research focus in recent two years [8-10].

Salivary adenoid cystic carcinoma (SACC) is one of most common malignancy of salivary gland, accounting for approximately 10% of salivary gland tumors and 30% of human salivary gland malignancy. Recurrence or/and early metastasis is its biological properties. SACC with high metastasis property occurs in all ages with a peak incidence of 40~60 years old and it is not sensitive to radiotherapy or chemotherapy. Clinical investigation showed that the incidence of SACC with distant metastasis, ranged from 35% to 50% of all cases, lung was the most common organ of its distant metastasis [11]. Distant metastasis of SACC cells often defeats the treatment of patients with SACC, and it is associated with a low long-term survival rate. Follow-up investigations have revealed that the lung metastasis is still the leading death cause of patients with SACC. At present, there is no any effective clinical therapy against the invasion and metastasis of SACC [12,13].

It has been proved that SACC is composed of two kinds of cells: neoplastic duct epithelial cells and neoplastic myoepithelial cells, the latter are the chief proliferative cells in SACC which could express salivary tumor markers such as smooth muscle actin, myosin, S-100 protein and GFAP, et al. The neoplastic myoepithelial cells secrete abundant proteoglycans full of the cribriform or tubular and glandular SACC [14,15]. A large number of studies have approved the close relationship between extracellular matrix and the biological features of SACC [14-16]. It has determined that extracellular matrix provides the essential microenvironment for the growth and metastasis of SACC. Being an important component of extracellular matrix, the abnormal proteoglycans play a very complex role in the carcinogenesis and metastasis of SACC. However, there is rare study of the relationship of proteoglycans and the metastasis behavior of SACC. The impact of proteoglycans on the metastasis mechanism of SACC is still unknown.

RNA interference (RNAi) is one of the most powerful gene-blocking technologies so far. RNAi can silence the expression of a special gene efficiently and rapidly by inhibiting the transcription product and down-regulate the protein expression of the target gene. A number of experimental studies have made it clear that RNAi could suppress the abnormal proliferation and metastasis of tumor cells. All these researches provided not only a good basis for the functional research but also a promising treatment of salivary gland tumors by using RNAi [17].

SACC-M derived from SACC-2 is a salivary adenoid cystic carcinoma cell line with high tendency of lung metastasis, and mainly has the differentiated and biological feature of the neoplastic myoepithelial cell. In this study, we constructed the short hairpin RNA (shRNA) expression vector targeting the coding regions of human XTLY-I gene to observe the relationship between SACC and proteoglycans. After the shRNA plasmid was introduced into SACC-M cells, the efficiency of XTLY-I gene suppression and proteoglycans reductoin were evaluated. Then the impact of down-regulated proteoglycans on cell migration and invasion was observed, the relationship between proteoglycans and the potential lung metastasis of SACC-M was analyzed as well.

The data of this research showed that the specific down-regulation of human XTLY-I by RNAi led to the decrease of proteoglycans in SACC-M cells; meanwhile the migration and invasion of SACC-M cells were inhibited significantly. Our research revealed that proteoglycans was a key factor in the process of invasion and metastasis of SACC. Thus, the suppression of proteoglycans may be an option for the treatment of SACC. The result of this research will also provide a new idea for RNAi usage in salivary gland tumor therapy.

## Methods

### Reagents and Cell lines

The human salivary adenoid cystic carcinoma cell line with high tendency of lung metastasis (SACC-M) was obtained from the Department of Oral and Maxillofacial Surgery, College of Stomatology, Shanghai Ninth People's Hospital Affiliated to Shanghai Jiaotong University. The cells were grown in Dulbecco's modified Eagle's medium (DMEM) (Hylone, Carlsbad, USA) containing 1500 mg/L glucose. All DMEM supplemented with 10% fetal bovine serum (Gibco, Gaithersburg, MD), penicillin and streptomycin at 37°C in a humidified atmosphere of 95% air and 5% carbon dioxide (NU-4750E, USA). The NIH3T3 cell line was kindly provided by Dr. Chao Wang (Hebei Medical University, P. R. China) and cultured in DMEM (Hylone, Carlsbad, USA) containing 4500 mg/L glucose and 1.5 mM l-glutamine supplemented with 10% fetal bovine serum.

Reagents were prepared and obtained from the following companies, Lipofectamine™2000 and TRIzol (Invitrogen, Karlsruhe, Germany); Blyscan Sulfated GAGs Assay (Biocolor, London, UK); SYBR Real-Time PCR premix Ex Taq Kit (Takara, Japan); RT-PCR Kit was from SBS Genetech co., Ltd. (Beijing, China). Rabbit anti-GAPDH polyclonal antibody, rabbit primary antibodies for XTLY-I and Pgenesil-1 vector (Genesil Biotechnology Co., Ltd., China) [18]. The enzymes of T4 DNA Ligase, *BamH*I, *Hind*III, *Sal*I, and PstI were from NEB Company (London, UK), Matrigel (Sigma, USA), Millicell chamber (with 8 μm diameter microspore, Millipore, USA), goat anti-rabbit horseradish peroxidase-conjugated secondary antibody (Santa Cruz Biotechnology) chemiluminescence reaction (Pierce, Rockford, IL). All BALB/C-nu mice used in this experiment received humane care. The nude mice were purchased from Laboratory Animal Center of Beijing Xiehe Medical University, P. R. China. Twenty 4-week-old female mice were chosen in this study. The animals were fed with certified standard diet and sterilized water in the condition of SPF with 25°C temperature and 30%~60% of humidity.

### The Design of shRNA and the Construction of Expression Vector

The human gene XTLY-I (Genbank accession number: NM_012688) was selected from GenBank. According to shRNA design principle [19], the shRNA sequence designed targeting nucleotide 2621-2642 of XTLY-I mRNA sequence is: AACAGGCAGCCCATCAAACCT. It was ensured that the selected shRNA sequence was different from other genes of human genome. EST database was searched by using BLAST http://www.ncbi.nlm.nih.gov/BLAST and no other isogenous genes were found. The negative control named shRNA-HK was designed and its sequence (GACTTCATAAGGCGCATGC) was not isogenous with any human gene. In order to overcome the disadvantages of short time action and lack of regenerating ability of small interfering RNA (siRNA)[20], the encoding shRNA plasmid expression vector was used with the U6 promoter and neo^r ^gene in this study. The hairpin loop of shRNA was TTCAAGAGA and the transcription terminator was four continuous T.

**Figure 1:**
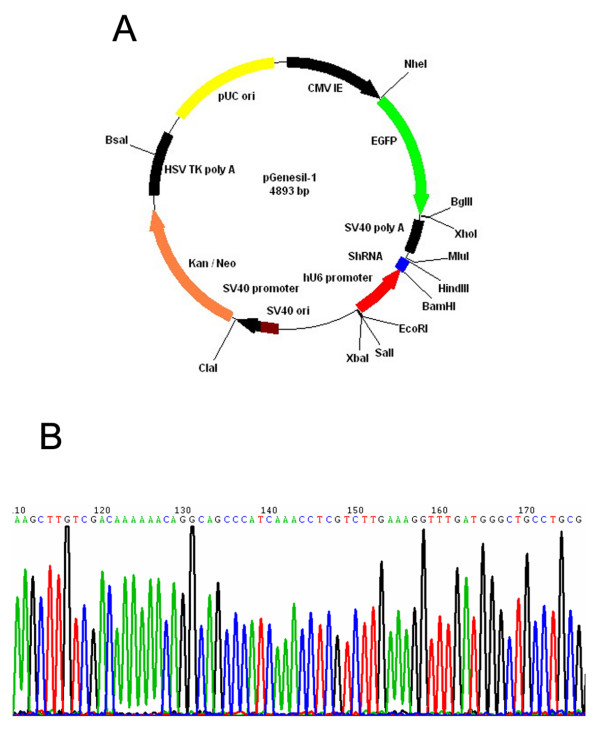
**Construction of shRNA vector targeting human XTLY-I gene**. A: The construct of Pgenesil-1 vector: This RNAi vector was a self-inactivating retroviral expression vector designed to express a small interfering dsRNA (siRNA) using the human U6 promoter. Neomycin-resistant selecting marker was used to select for stable clones. GFP was used to determine the successful transfection. B: shRNA-WJ4: AGCTTGTCGACAAAAAACAGGCAGCCCATCAAACCTCGTCTTGAAA G GTTTGATGGGCTGCCTGCG.

The shRNAs were subcloned into the Pgensil-1 with human U6 promoter (Fig. 1A), between the *Sal*I and XbaI restriction sites. All of the constructs used in this study were verified by DNA sequencing. Once the requirement had been met, a large-scale preparation of plasmid DNA was extracted; the DNA plasmids were named shRNA-WJ4 (the RNAi plasmid), shRNA-HK (the nagetive control plasmid), respectively. The structure of the shRNA vector is:*BamH*I + Sense + Loop + Antisense + ending signal + *Sal*I + *Hind*III [21].

### Cell Transfection and Generation of Stable Cell Line

Twenty-four hours before transfection, SACC-M cells were plated in a 6-well plate with 1 × 10^5 ^cells per well until the cells reached the 70%~80% confluence at next day. They were washed twice by DMEM (containing 1500 mg/L glucose), then 2 ml DMEM free of fetal bovine serum and antibiotics was added into each well. The cells were transfected according to the manufacturer's protocol of cationic lipid complexes Lipofectamine™2000. SACC-M cells transfected with DMEM medium lacking DNA plasmid served as the blank control. SACC-M cells transfected with shRNA-HK served as the negative control. The 4.0 μg DNA plasmid and 10 μl Lipofectamine™ 2000 was diluted by 250 μl DMEM, respectively. Then being mixed and stayed at room temperature for 15~20 min, the compound was added to each well and thoroughly mixed with medium. Fresh growth medium with fetal bovine serum was replaced after 6 hours of transfection. Efficiency was evaluated after 48 hours of transfection by observing the green fluorescent protein (GFP) expression under the fluorescence microscope (Olympus PM-10A, Japan).

After 48 hours of transfection the stable transfectant cells were acquired by using G418 (500 μg/ml). Culture medium was replaced at least twice per week. A stable cell pool was selected after addition of G418 for about 3 weeks. Visible cell clones were picked up and expanded. The stable transfectant cells expressing green fluorescence were observed under microscope, however the cells without shRNA plasmid did not show any green fluorescence. Three groups included in this study were: group SACC-M: the normal cultured SACC-M cells; group SACC-M-HK: transfected with the negative control plasmid shRNA-HK; group SACC-M-WJ4: transfected with shRNA-WJ4.

### Real-Time Quantitative PCR for XTLY-I mRNA Expression

The genotypes of the stable G418 resistance cells SACC-M-WJ4 and SACC-M-HK were detected by real-time quantitative PCR. Total RNA was extracted from SACC-M-WJ4, SACC-M-HK and SACC-M cells by using Trizol reagent as described in the manufacture's instruction. The production of cDNA was amplified by using gene-specific primers (shown in Table 1). Real-time quantitative PCR was performed by GenAmp PCR system 2700 (AB Company, USA) and SYBR Green I Real-Time PCR premix Ex Taq Kit following the manufacture's protocols. The results were analyzed by using the Light Cycle Real-time PCR System (Switzerland Roche Company) to obtain Cycle threshold. Finally, the computer analyzed the fluorescence signal automatically. All quantitative data were normalized to an endogenous control GAPDH. The relative quantitative value of each targeting gene was analyzed by using a comparative copy number. The mRNA expression of target gene in each cycle was obtained [22].

**Table 1:** Real-time PCR primers

Gene	Sequence	Product Sizes
XTLY-I	5'-GCCTGGAAAGTGATAGCACTAAA-3' 5'-GGAGCAATGGAAGGGTAAAGA-3'	106 bp
GAPDH	5'-CATGAGAAGTATGACAACAGCCT-3' 5'-AGTCCTTCCACGATACCAAAGT-3'	113 bp

### Western Blot Analysis for XTLY-I Protein Expression

The protein expression of XTLY-I in SACC-M-WJ4 cells and SACC-M-HK cells was detected by Western blot, and then compared with SACC-M cells. Cells were seeded in 100 cm^2 ^flasks. Twenty-four hours after plating, confluent cell layers were washed with ice-cold PBS and lysed for 30 min at 4°C with 1% NP-40, 0.1% Triton X-100, 30 mM sodium phosphate (pH 7.4) containing 1 mM sodium orthovanadate, 2.5 mM Tris-HCl (pH 7.5), 100 mM NaCl and 10 μg/mL of leupeptin, aprotinin. Then, the homogenate was centrifuged at 12,000 g for 20 min at 4°C. The supernatant was collected and protein was quantified with the Bio-Rad protein colorimetric assay. After addition of sample buffer to the cellular extract and boiling samples at 95°C for 5 min, protein was separated on 8% SDS-PAGE gel. Protein was transferred onto PVDF membrane (Millipore Corporation, Bedford, MA) and the membrane was blocked for 1 h at room temperature with 5% BSA (bovine serum albumin) in Tris-buffered saline containing 0.05% Tween 20 (TBST). Next, blots were washed and incubated overnight at 4°C in TBST containing 1% BSA with rabbit primary antibodies for XTLY-I (1: 1000) and rabbit anti-GAPDH (1: 2000). Membranes were washed three times with TBST, incubated with goat anti-rabbit horseradish peroxidase-conjugated secondary antibody (1: 2500 dilution in TBST containing 1% BSA) for 120 min at room temperature and then washed three times with TBST. After chemiluminescence reaction, bands were detected by exposing the blots to X-ray films for the appropriate time period. For quantitative analysis, bands were detected and evaluated densitometrically with Foretix ID software, normalized for GAPDH density.

### Quantitation of Proteoglylcans (Total GAGs)

The cell culture supernatant of the three groups was collected and the content of total GAGs, which is the major component of proteoglycans, was measured. The total GAGs were quantitated using the Blyscan™ Sulphated Glycosaminoglycan Assay Kit according to the manufacturer's instructions. The assay was based on a colorimetric reaction between the negatively charged GAGs and the positively charged dye dimethylmethylene blue (DMB) [23,24]. Concentration was determined from the standard curves of chondroitin sulfate A. Meanwhile the corresponding cells were digested by trypsin and counted carefully. The content of proteoglycans secreted by 1 × 10^6 ^cells was detected at different time of 24, 48, 72, 96 and 120 hours, respectively.

### Cell Adhesion to Matrigel

The 96-well plates were coated with Matrigel according to the producer's guideline. Cells including SACC-M-WJ4 cells, SACC-M cells and SACC-M-HK cells were seeded onto the Matrigel-coated wells (1 × 10^4 ^cells per well), respectively. After being incubated at 37°C with complete medium (pH 7.2) for 1 hour, the wells were washed and the cells adhere to Matrigel firmly were lysed in 20 μl MTT for 4 hours at 37°C. Then 150 μl DMSO was added into each well and spectrometric absorbance was measured at the wavelength of 490 nm, the cell adhesion ratio was determined as follow: (value of experimental group/value of blank control - 1) × 100%. Each experiment was performed at least three times independently.

### Matrigel Invasion Assay

Cell invasiveness was determined in vitro to evaluate the ability of SACC-M cell to transmigrate a layer of reconstructed extracellular matrix (Matrigel). Three group cells of SACC-M-WJ4, SACC-M and SACC-M-HK (3 × 10^5 ^cells in 1 ml of DMEM containing 0.1% BSA) were assayed in triplicate for Matrigel invasion at 37°C in a humid incubator (95% air, 5% CO_2_) for 24 hours by using Millicell chambers (Millipore, MD), respectively. The polycarbonate filters (13 mm in diameter, 8-μm pore size; Costar Scientific, Cambridge, MA) were coated with Matrigel as described in the introduction. DMEM medium conditioned by NIH3T3 fibroblasts was used as a chemoattractant and added into the lower compartment of the Millicell chamber. DMEM containing 1% serum and 0.1% BSA was added into the upper compartment of the Millicell chamber instead of the chemoattractant. After 24 hours incubation, the filters were removed and fixed with methanol for 15 min; the cells on the upper side of the membrane were removed. Invasive cells, which were able to breach 8 μm pores and grew on the lower side, were stained with hematoxylin and eosin, the cells migrating to the lower side of the PVPF membrane were counted under the inverted microscope (Olympus PM-10A, Japan) in 5 fields selected randomly (100× magnification). Each experiment was repeated three times.

### Wound Healing Assay

Wound healing assay was carried out to determine the cell protrusion and migration ability of tumor cells. SACC-M cells and their derivative cell lines were seeded into 6-well dishes and grew until 80% - 90% confluence. Sterilized one-milliliter pipette tip was used to generate wounding across the cell monolayer, and the debris was washed with PBS. Migration of cells into the wound was then observed at different time. Cells migrated into the wounded area or protruded from the border of the wound were visualized and photographed under the inverted microscope at different time. A total of nine areas were selected randomly in each well by a 100× magnification and cells in three wells of each group were quantified in each experiment. Experiments were carried out in triplicate at least three times.

### Experimental Lung Metastasis

Twenty 4-week-old female BALB/C nude mice were randomized into 4 groups of 5 mice each. The tail veins of nude mice were injected with 0.2 ml of cell suspension containing 2 × 10^6 ^cells of SACC-M-WJ4, SACC-M-HK and SACC-M, respectively, and normal saline (N.S) instead of tumor cells was injected into the tail veins acted as normal control. The nude mice were sacrificed at the 6^th ^week after injection. The fresh lung samples were harvested and weighed; the metastasis rates and the number of metastasis nodules on the lung surface were detected. The lung samples were used for histopathology analysis. Formalin-fixed/paraffin-embedded specimens were prepared by ordinary procedures, 4 μm thick sections were stained with hematoxylin and eosin, and then examined under the microscope (Olympus AX-80, Japan) to evaluate the morphological changes of metastasis tumors. The size and metastasis grading were detected.

### Statistical Analysis

Software SPSS 10.0 was used for all statistical evaluation. All data were expressed as mean ± standard error. Data were analyzed for statistical significance by using one-way ANOVA. *P *< 0.05 was considered statistically significant.

## Results

### Construction of shRNA vector targeting human XTLY-I gene and the isolation of clones stably expressing shRNA targeting XTLY-I

**Figure 2:**
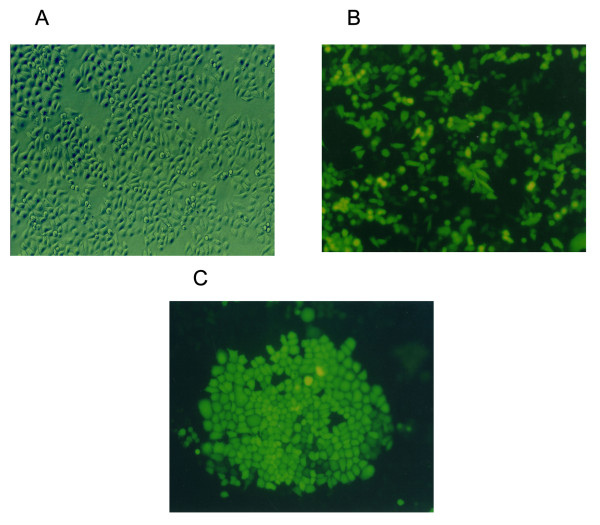
**SACC-M cells and the cell clone (A-C)**. (A): The SACC-M cells. (The inverted microscope, × 100); (B): Forty-eight hours after transfection with shRNA-WJ4 expressed green fluorescent protein (The inverted fluorescent microscope, × 100); (C): A cell clone of SACC-M-WJ4 selected with G418 (The inverted fluorescent microscope, × 100).

The plasmid Pgenesil-1 has *Pst*I digestion sites. After the insertion of segments of target gene template DNA, the *Pst*I digestion sites were replaced. In the inserted target gene template DNA, a *Sal*I digestion site was designed between *BamH*I and *Hind*III of Pgenesil-1. Once it was correctly inserted, *Sal*I produced a 400 bp DNA segment and it was correctly identified. The bacteria solution of transformation was tested by Shanghai Yingjun Company with the results in agreement with the design. The eukaryotic expression vector of shRNA targeting human XTLY-I gene was constructed and named shRNA-WJ4. The negative control plasmid was named shRNA-HK. Successful constructions were identified by digestion and sequencing analysis (Fig. 1B). The plasmid shRNA-WJ4 and negative control shRNA-HK was transfected into SACC-M cells, respectively. SACC-M cells transfected with the plasmids expressed green fluorescent successfully. Transfection efficiency (≈ 70%) was evaluated after 48 hours of transfection (Fig. 2B). After G418 selection for about 3 weeks, the stable sub-clone cells named SACC-M-WJ4 (expressed shRNA-WJ4) were isolated and pooled together for further analysis (Fig. 2C). The negative control SACC-M-HK cell expressed shRNA-HK was isolated by the same method and no obvious change in cell morphology was observed in comparison with the parental cells (Fig. 2A). These clones were used for the subsequent experiments

### ShRNA expressing vector inhibited XTLY-I gene and proteoglycans secreted by SACC-M cells decreased

**Figure 3:**
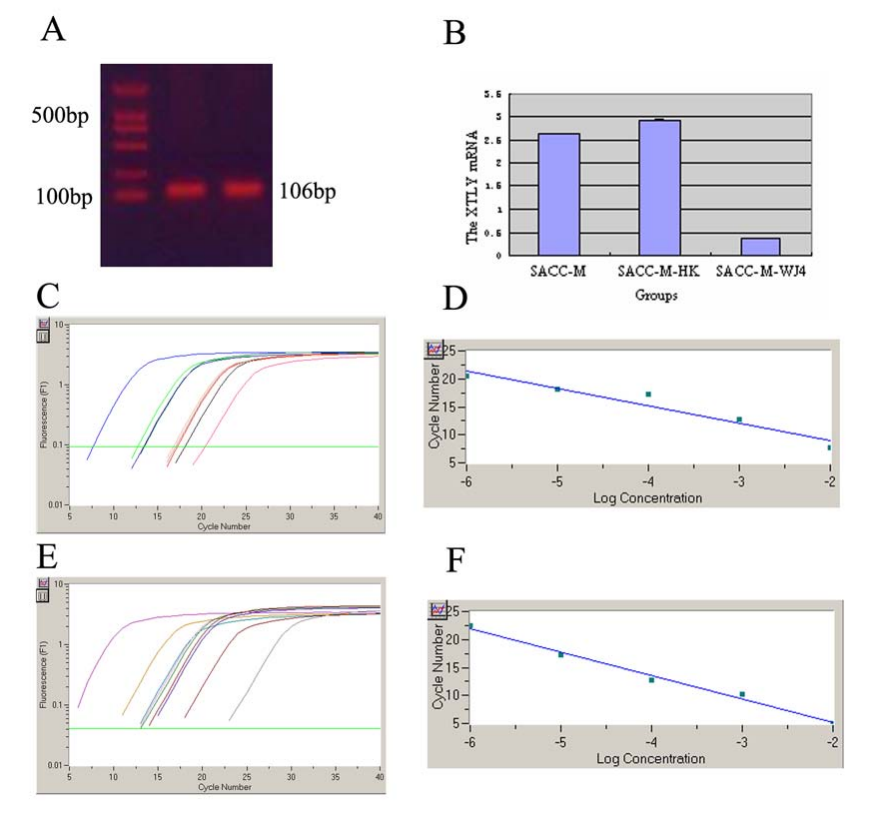
**The results of Real-Time PCR (A-F)**. (A) The amplification fragment of XTLY-I gene; (B) The XTLY-I mRNA expression in different groups: Group SACC-M-WJ4 compared with that of group SACC-M and SACC-M-HK (*P *< 0.05); (C) The fluorescent quantitative curve of positive standard samples of XTLY-I gene; (D) The fluorescent quantitative standard curve of XTLY-I gene; (E) The fluorescent quantitative curve of standard samples of GAPDH; (F) The fluorescent quantitative standard curve of GAPDH.

**Figure 4:**
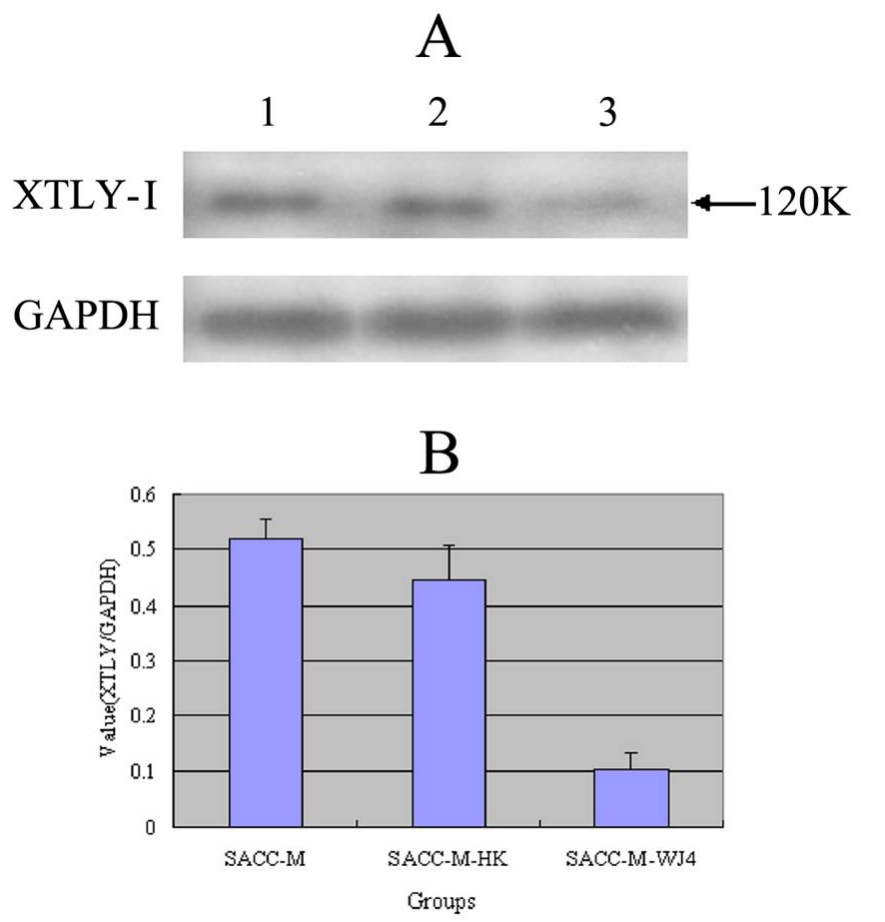
**The protein expression of XTLY-I by Western blot analysis**. (A): Detection of XTLY-I protein expression in different groups by Western blot analysis. Band of XTLY-I (120 kDa.) was identified in different cells. Lane 1: SACC-M cells; lane 2: SACC-M-HK cells; lane 3: SACC-M-WJ4 cells. Fig. 4 (B): The comparison of XTLY-I protein expression in different groups. Value (XTLY/GAPDH) means the band integrated opitical density (IOD) ratio of XTLY and GAPDH.

In order to detect the silence efficiency of shRNA expression vector targeting XTLY-I gene, real-time PCR was performed to detect the mRNA expression. The protein expression of XTLY-I was detected by Western blot analysis as well in the stably transfected cells and their parental cells. The results showed that the XTLY-I mRNA of SACC-M-WJ4 cells was 0.381 consistent with the protein expression levels (0.103) (Fig. 3A-F & Table 2). The results of Western blot demonstrated clearly that SACC-M-WJ4 cells showed a dramatic decrease in the expression of XTLY-I protein (Fig. 4A-B & Table 2). The RNAi plasmid shRNA-WJ4 showed powerful RNAi effect compared with the two controls. The mRNA expression of SACC-M-WJ4 decreased by 86.81%, and the protein expression was decreased by 80.10%, respectively. The data demonstrated that DNA-based shRNA vector constructed in this study was effective in XTLY-I gene silence in SACC-M-WJ4 cells.

**Table 2:** The expression of mRNA and protein of XTLY-I gene (mean ± SD)

Value	SACC-M	SACC-M-HK	SACC-M-WJ4
mRNA	2.609 ± 0.001	2.898 ± 0.025	0.381 ± 0.000
Protein	0.518 ± 0.036	0.446 ± 0.062	0.103 ± 0.030
Statistic result*	A	A	B

*The same letters in the statistical results between the two groups showing no significant difference, while different letters showing significant differences.

**Figure 5:**
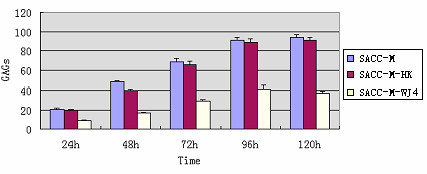
**The GAGs contents of the three groups in different time (μg/10^6 ^cells)**.

Then the level of proteoglycans (the total GAGs) secreted by the three group cells was tested by biocolor method. Overall, the GAGs secreted by the three kinds of cells from 24 hours to 96 hours were much more than that of 96 hours to 120 hours. The detail data showed that the effective silence of human XTLY-I gene reduced the secretion of GAGs dramatically in SACC-M-WJ4 cells as shown in Table 3 and Fig. 5. Compared with the parental SACC-M cells and SACC-M-HK cells, the total GAGs selected by SACC-M-WJ4 cells decreased significantly during the whole experimental period, there was significant differences between SACC-M-WJ4 cells and SACC-M cells (*P *< 0.05); There was no significant difference between SACC-M-HK cells and SACC-M cells at the secretion of GAGs (*P *< 0.05). The total GAGs inhibitory rate of SACC-M-WJ4 cells was 58.17% (24 h), 66.06% (48 h), 57.91% (72 h), 59.36% (96 h), and 55.65% (120 h) respectively, as shown in Table 4. These data indicated that the silence of human XTLY-I gene was responsible for the suppression of proteoglycans secretion and biosynthesis in SACC-M-WJ4 cells.

**Table 3:** The total GAGs content in each group (μg/10^6 ^cells) (mean ± SD, n = 3)

Times	SACC-M	SACC-M-HK	SACC-M-WJ4
24 h	20.56 ± 1.03	19.85 ± 0.84	8.60 ± 0.94
48 h	48.71 ± 1.07	39.97 ± 1.34	16.53 ± 0.89
72 h	68.73 ± 3.23	65.19 ± 3.56	28.93 ± 1.34
96 h	91.0 ± 3.06	88.64 ± 3.52	41.56 ± 3.82
120 h	93.70 ± 3.08	91.21 ± 2.89	36.98 ± 2.47

**Table 4:** The inhibitory rate of total GAGs contents (%)

Group	24 h	48 h	72 h	96 h	120 h
SACC-M-HK	3.45	17.94	5.15	2.59	2.66
SACC-M-WJ4	58.17	66.06	57.91	59.36	55.65

### Down-regulated proteoglycans inhibited the ability of adhesion and metastasis of SACC-M cells

**Figure 6:**
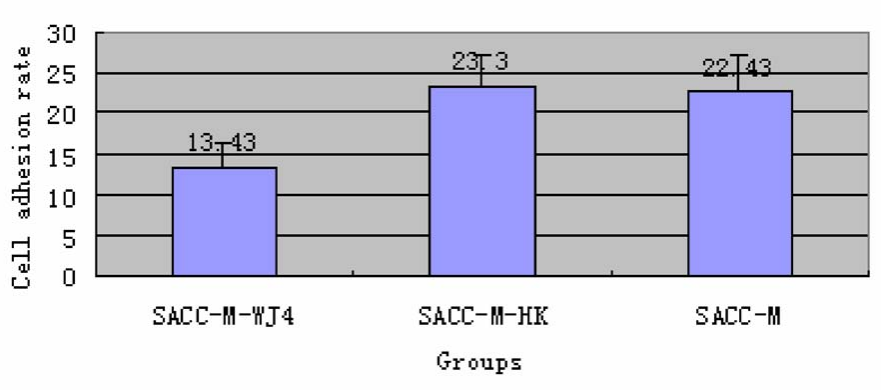
**The comparsion of cell adhesion rate of three groups**.

It was generally believed that extracellular matrix molecules were involved in tumor metastasis and proteoglycans contained lots of cell adhesion factors act as important regulators in this metastasis. Cell adhesion to extracellular matrix is a preliminary step during the invasion and metastasis of tumor cell. Therefore, the effect of down-regulated proteoglycans on tumor invasion was investigated by observing the change of cell adhesion. The detection of cell adhesion of SACC-M, SACC-M-HK and SACC-M-WJ4 cells was performed by the adhesion assay. The adhesion rate of group SACC-M -WJ4 was 13.43 ± 2.91%, much lower than that of group SACC-M (23.3 ± 3.96%) and group SACC-M- HK (22.43 ± 4.85%), *P *< 0.05. On adhesion assays, SACC-M-WJ4 cells showed a 42.36% decrease in binding to Matrigel compared with that of the negative control. There was no significant difference between SACC-M-HK and SACC-M cells (shown in Fig 6). The data demonstrated that reduction of proteoglycans could indeed decrease the ability of cells to adhere to extracellular matrix molecules.

### Suppression of proteoglycans decreased the ability of Matrigel transmembrane migration of SACC-M cells

**Figure 7:**
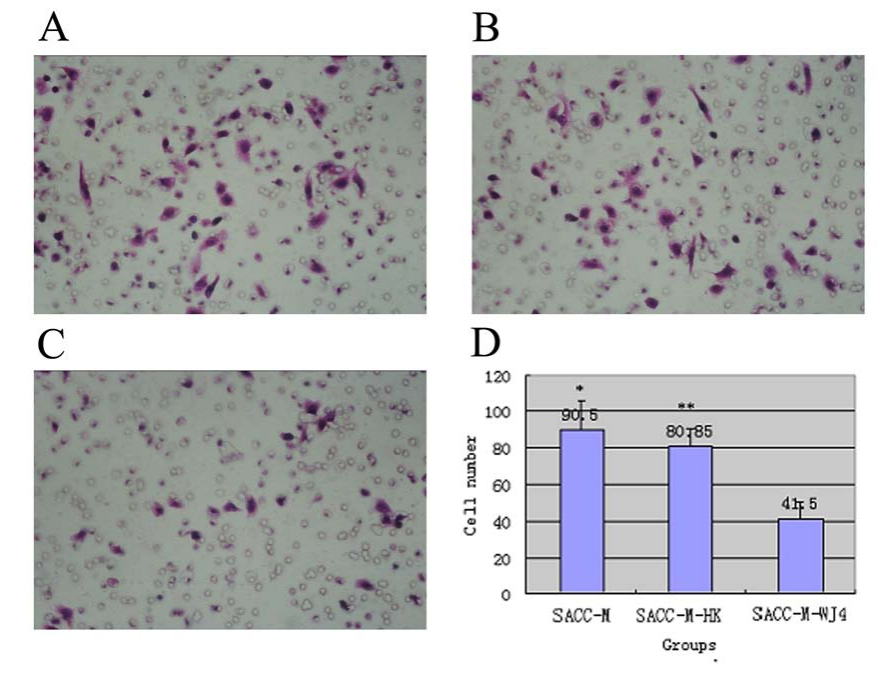
**Suppression of proteoglycans hampered transmembrane migration ability of SACC-M-WJ4 cells**. (A): SACC-M cells migrating to the lower side of membrane coated by Matrigel; (B): SACC-M-HK cells migrating to the lower side of membrane coated by Matrigel; (C): SACC-M-WJ4 cells migrating to the lower side of membrane coated by Matrigel; (D): Compared with group SACC-M and group ACC-M-HK, the invading ability of SACC-M-WJ4 reduced by over 50%.

Invasion of basement membranes is thought to be a critical event in tumor metastasis. It is known that proteoglycans secreted from the tumor cells was not only responsible for the formation of basement membranes, but also mediate the invasion of tumor cells. This process is best evaluated in vitro by the transmigration of a biologically active matrix such as Matrigel. Therefore, we measured the ability of these cells to transmigrate through the Matrigel membrane. Significant differences in cell invasion were observed in this analysis. As shown in Fig. 7: A-D, under the same condition of incubating, SACC-M-WJ4 cells display a significantly lower transmembrane migration activity (Fig. 7C). The transmembrane cell of group SACC-M-WJ4 cells was 41.50 ± 8.35; much lower than that of group SACC-M-HK (83.53 ± 10.21) (Fig. 7A) and group SACC-M cells (90.50 ± 15.28) (Fig. 7B), *P *< 0.05; the transmembrane cells of group SACC-M-WJ4 was decreased by 54.14% compared with that of the negative control. There was no significant difference between SACC-M-HK and SACC-M cells. These data showed that SACC-M-WJ4 cells with down-regulated proteoglycans could inhibit the ability of their invasion through Matrigel.

### Suppression of proteoglycans secretion decreased cell mobility

**Figure 8:**
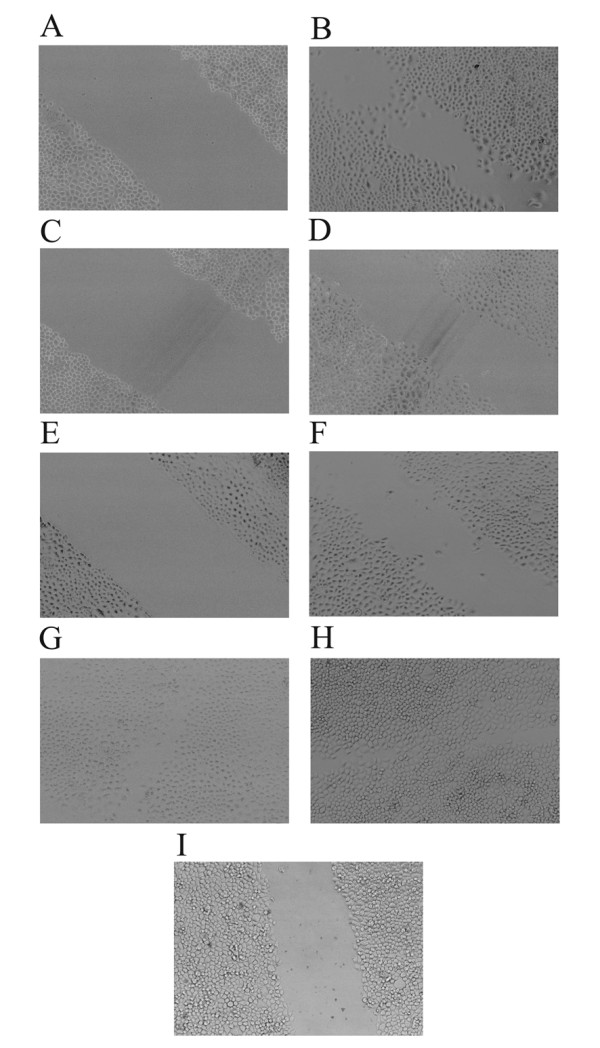
**Suppression of proteoglycans decreased the migration of SACC-M-WJ4 cells**. (A): Wound healing assay of group SACC-M (0 h); (B): Wound healing assay of group SACC-M (12 h); (C): Wound healing assay of group SACC-M-HK (0 h); (D): Wound healing assay of group SACC-M-HK (12 h); (E): Wound healing assay of group SACC-M-WJ4 (0 h); (F): Wound healing assay of group SACC-M-WJ4 (12 h); (G): Wound healing assay of group SACC-M (48 h); (H): Wound healing assay of group SACC-M-HK (48 h) (I): Wound healing assay of group SACC-M-WJ4 (48 h).

The metastatic potential of tumors depends on the ability of tumor cell to invade and migrate to distant sites. Active cell motility is another important step of tumor cell invasion. The mobility of cells was measured by using a well-established wound healing assay in vitro. The results showed that after 12 hours of scraping, the cells of group SACC-M-WJ4 migrating into the wounding region was 115.92 ± 6.81, much less than that of group SACC-M-HK (283.53 ± 9.72) and group SACC-M (289.50 ± 23.02), such changes were statistically significant, *P *< 0.01. There was no significant difference between SACC-M-HK and SACC-M cells, as shown in Fig 8: A-I & Table 5.

**Table 5:** The results of wound healing assay (Mean ± SD)

Group	Cell numbers
SACC-M	289.50 ± 23.02 *
SACC-M-HK	283.53 ± 9.72**
SACC-M-WJ4	115.92 ± 6.81

*compared with group SACC-M-WJ4: *P *< 0.01;

**compared with group SACC-M-WJ4: *P *< 0.01;

*compared with **: *P *> 0.05.

### Reduction of proteoglycans decreased the lung metastasis of SACC-M cells

**Figure 9:**
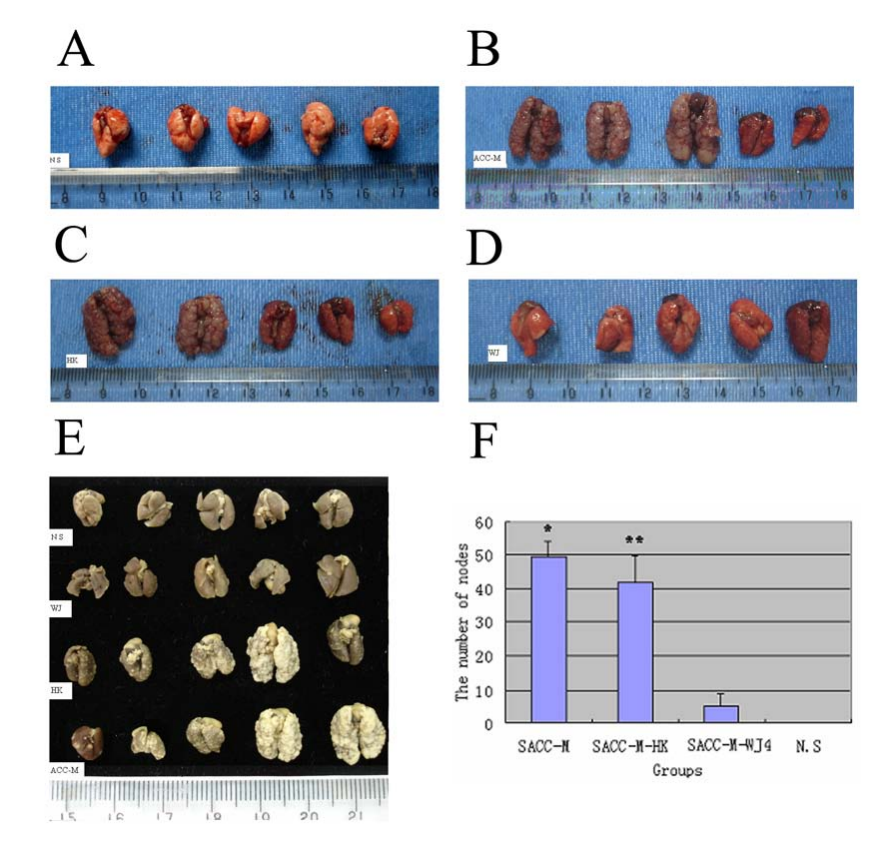
**Lung metastasis samples of different groups (A-E)**. 9 (A) The normal lung of group N.S without metastatic tumor; (B) The lungs of group SACC-M with many metastatic tumors; (C) The lungs of group SACC-M-HK full of metastatic tumors; (D) The lungs of group SACC-M-WJ4 with few metastatic tumors; (E) The lung samples of the different group fixed with Formalin. (F) The number of metastasis node on lung surface of different group.

We also tested the effects of down-regulated proteoglycans on the potential metastasis of lung. SACC-M-WJ4, SACC-M-HK and SACC-M cells were injected into the tail veins of nude mice, respectively. The results showed that the parental SACC-M cells resulted in the extensive lung metastases (Fig. 9B), whereas the inhibition of proteoglycans synthesis in SACC-M-WJ4 cells reduced the lung metastasis significantly (Fig. 9D). The lung metastasis of group SACC-M-WJ4 was decreased to 40% (2/5), significantly lower than that of group SACC-M-HK (100%) (Fig. 9C) and group SACC-M (100%), *P *< 0.05. The number of metastasis nodes on the surface of lung was 49.4 ± 4.81 in group SACC-M, 41.6 ± 8.09 in group SACC-M-HK and 4.8 ± 3.89 in group SACC-M-WJ4, respectively, *P *< 0.01(Fig. 9E-F). The lung wet weight of group SACC-M-WJ4 (0.226 ± 0.83 g) was significantly lower than that of group SACC-M (0.897 ± 0.21 g) and group SACC-M-HK (0.786 ± 0.28 g), *P *< 0.01, shown in Table 6; there was no significant difference between group SACC-M-WJ4 and group N.S (0.196 ± 0.03 g) (Fig. 9A &9D). This suggested an important role of proteoglycans in the metastasis of SACC-M cell (Table 6). Multinodular lesions on the surface and inside of lung in group SACC-M-HK and group SACC-M were observed, the sizes were from miliaria to pea, some were larger to integrate several protuberances on the surface. In group SACC-M-WJ4 only two mice had lung metastasis with miliary nodules (Fig. 9E, &10).

**Table 6:** Lung weight with metastatic nodes (Mean ± SD)

Group	Wet weight (g)
SACC-M	0.897 ± 0.21*
SACC-M-HK	0.786 ± 0.28**
SACC-M-WJ4	0.226 ± 0.83
N.S	0.196 ± 0.03

*compared with group SACC-M-WJ4 and group NS, respectively: *P *< 0.01; **compared with group SACC-M-WJ4 and group NS, respectively: *P *< 0.01; *compared with **: *P *> 0.05;

group SACC-M-WJ4 compared with group N.S: *P *> 0.05.

**Figure 10:**
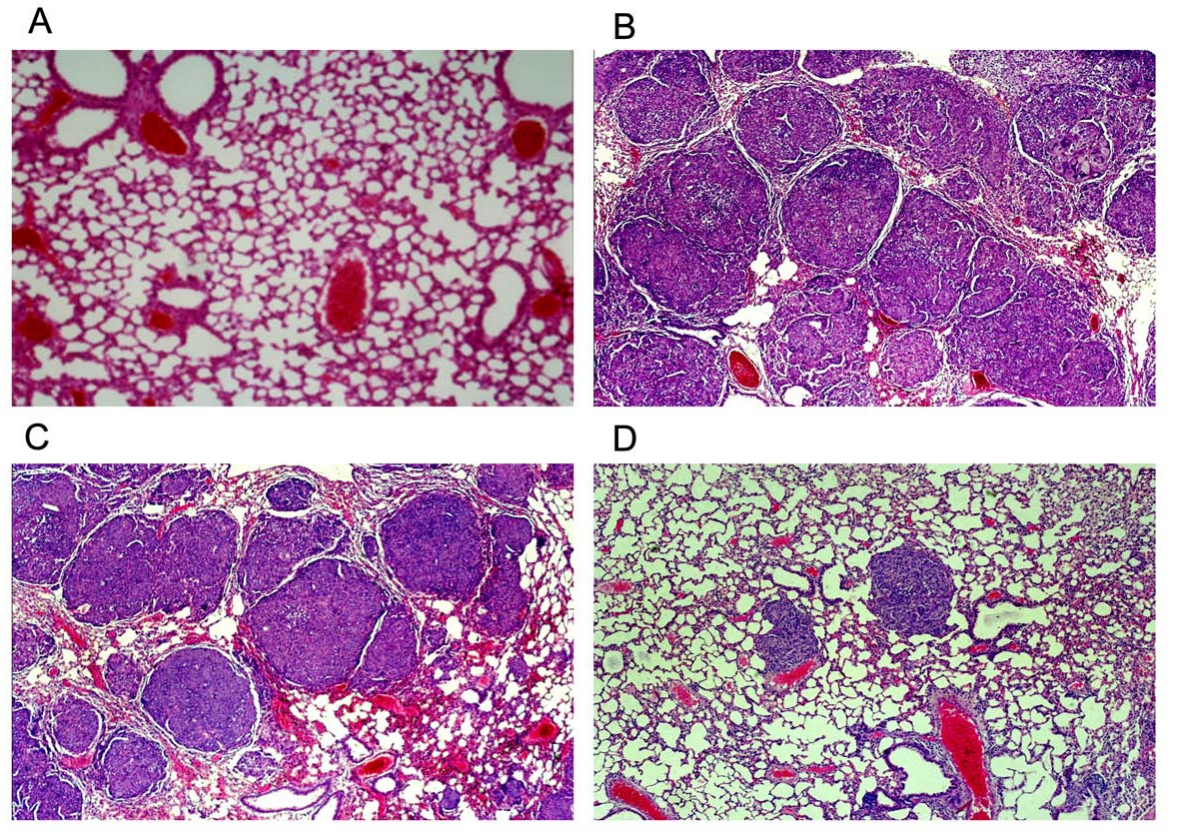
**Histological observation of the lung in four groups (A-D)**. (A): The normal lung of group NS (HE × 100); (B) Many metastatic nodes in the lung of group SACC-M (HE × 100); (C) Many metastatic nodes in the lung of group SACC-M-HK (HE × 100); (D) Only a few smaller metastatic nodes in the lung of group SACC-M-WJ4 (HE × 100).

The histopathological analysis of the lung samples showed that no lung metastasis was found in group N.S, and the normal structure of lung could be observed clearly (Fig. 10A). The normal structures of lungs in group SACC-M (Fig. 10B) and group SACC-M-HK (Fig. 10C) were almost destroyed. The lungs were full of metastasis nodules with different sizes. The tumor cells were round or polyhedral with eosinophilic cytoplasm and round nucleus, arranged in dense clumps of solid, many mitoses could be observed. Pulmonary vascular cavities were in condition of hyperaemia. Only a few metastasis nodules were found in the two lung samples of group SACC-M-WJ4 and the normal structure of them was still remained (Fig. 10D). The size of the metastasis nodule in group SACC-M-WJ4 was much smaller than that of group SACC-M and group SACC-M-HK, wrapped by an incomplete capsule. Some of them were only cell lumps. The tumor cells arranged loose with less mitosis. These data showed that reduction of proteoglycans decreased the lung metastasis of SACC-M cells.

## Discussion

As a type II transmembrane proteins localized in Golgi apparatus, human XTLY-I is a member of glycosyltransferase family 14, whose systematic name is UDP-α-D-xylose: proteoglycan core protein β-D-xylosyltransferase. It has determined that XTLY-I catalyzes the initial step in which GAGs chains were attached to the core protein. This step is rate-limited and is thought to be the most important regulator in proteoglycans biosynthesis [6,25]. Recent advances in mammalian have showed that XTLY-I activity is a biochemical marker for the assessment of proteoglycan-associated diseases [26], such as systemic sclerosis, diabetic nephropathy, osteoarthritis, pseudoxanthoma elasticum (PXE), etc. The studies pointed out the important role of XTLY-I as a disease modifier in pathologies with an altered proteoglycans metabolism. In vivo in salivary gland, the acinus and duct cells do not produce and secrete proteoglycans, so they do not express proteoglycans and XTLY-I. Recently years, the research of Jie W. et al. had demonstrated that the normal myoepithelial cells do not synthesize and secrete proteoglycans, but when they transform to tumor cells, the neoplastic myoepithelial cells in salivary gland tumors, they have the ability to synthesize and secrete proteoglycans abnormally [14,27]. The similar results had been also demonstrated by other researches [28,29]. As the crucial impact of XTLY-I on proteoglycans synthesis and in order to determine the role of proteoglycans in the metastasis of human SACC, XTLY-I gene was blocked by shRNA expression vector to suppress the biosynthesis of proteoglycans in SACC-M cells. This study showed that the biosynthesis and secretion of proteoglycans could be supressed in tumor cells by the silence of XTLY-I.

RNAi in mammalian is to inhibit the function of specific gene by introducing siRNA or siRNA expression vector with high silent efficiency into subjects (such as cells, tissues, etc.). For effective gene silence mediated by RNAi, vector plasmids have been constructed to express siRNA as inverted repeats showing similar potency to initial RNAi. These siRNA-like molecules are commonly designated as shRNA. For generating shRNAs, these vectors have been designed to be able to obtain the stable expression of shRNA by using strong RNA polymerase-II-dependent promoters like CMV, or RNA polymerase-III promoters like U6 or H1. As RNA polymerase-III expression systems can offer a great potency for maintaining stable expression of short RNA molecules in vitro as well as in vivo [20], the U6-driven expression vector Pgensil-1 was applied in this study. The size of the loop structure of shRNA has a considerable influence on the gene suppression activity. A mounting research reported that a 9-nucleotide loop showed more effects than a 7-nucleotide or 5-nucleotide loop [30-32]. Therefore, the shRNA was designed with a 9-nucleotide loop in this study.

The DNA-based vector plasmid shRNA-WJ4 in this study showed powerful RNAi efficiency, whereas the negative control shRNA-HK did not show any significant RNAi effect on SACC-M cells. When mRNA and protein levels of XTLY-I were measured simultaneously, there was a good match between RNA transcript loss and protein loss, indicating that the primary mechanism of gene knockdown was RNA transcript-mediated knockdown, which required a high degree of sequence homology between the siRNA and target sequences.

After the silence of XTLY-I gene, the cells could not synthesize the following GAGs chain, which attached to the core protein of proteoglycans, then the biosynthesis of proteoglycans could not be achieved [33]. As proteoglycans were secreted mainly into the extracellular matrix, the total GAGs in cell culture medium were the proteoglycans secreted by SACC-M cells indeed. The data of this research showed that the silence of human XTLY-I gene resulted in the inhibition of proteoglycans secretion. The total GAGs secreted by SACC-M-WJ4 cells reduced dramatically during the experimental times. Meanwhile, we also transfected SACC-83 cells with shRNA-WJ4 plasmid, the reduction of total GAGs was observed clearly (data not shown). The data demonstrated that the expression of XTLY-I gene was related to proteoglycans level closely.

Invasion and metastasis are the major biological characteristics of SACC, and also an important indicator of malignancy. Extracellular matrix plays an important and necessary role in the process of tumor invasion and metastasis. Extracellular matrix mainly consists of two compounds: basement membrane and connective tissue. Since most proteoglycans such as heparan sulphate proteoglycan (HSPG), keratan sulphate proteoglycan (KSPG), chondroitin sulfate proteoglycans (CSPG) etc. express on the surface of cytoplasmic membrane and deposit in the extracellular space, they are not only the major component of mesenchyme but also the main component of basement membrane. The secretion level and the type of proteoglycans impact the function of extracellular matrix in tumor to a great degree [1].

SACC is the most characteristic malignancy of oral and maxillofacial regions. Previous studies showed that neoplastic myoepithelial cells in SACC secreted different proteoglycans including HSPG, KSPG, CSPG and hyaluronan (HA), etc. Proteoglycans, which showed complex effect on tumor invasion and metastasis, were closely related to the invasion and metastasis of SACC [28,34,35]. SACC histologically characterized by a cribriform appearance resulted from multi-pseudocystic spaces. Evidences indicated that extracellular matrix molecules and their receptors played important role in the morphogenesis of SACC. Clinical data showed that SACC with cribriform pattern had a better prognosis than those with a solid pattern. There were more patients with solid pattern occurring early recurrence and distant metastases. Although SACC patients with a cribriform pattern rich in proteoglycans had less distant metastasis, they underwent local recurrence more easily. However, solid SACC lack of proteoglycans had the worst survival rate in all kinds of SACC [29,35-38]. It seemed to imply that abundant proteoglycans in extracellular matrix could suppress the invasion of SACC. SACC-M cells are highly invasive and tumorigenic isolated from a SACC cell line SACC-2 that derived from human minor salivary gland in palate. Some scholars had compared the proteoglycans expression in SACC-M cell and its parental cell SACC-2; the expression of proteoglycans was significant higher in SACC-M cells with a high potential metastasis. The speed of proteoglycans biosynthesis and deposition in the extracellular matrix was significantly increased than that of SACC-2 cells [39]. Kimuras et al. [29] had found in their study, the ability of cell adhesion and growth was enhanced significantly when the cells were cultured in the media rich of proteoglycans. These researches supported that the existence of sufficient proteoglycans promoted the metastasis of tumor.

The metastasis of tumor is a continuous process involved in a variety of factors with homologous target. The basic process of metastasis includes that tumor cells separate away from the surrounding cells and migrate to the extracellular matrix, then adhere to the basement membrane and degrade them; simultaneously the cells infiltrate the vascular and get to the target organ by degrading the wall proteins. With the rapid proliferation of tumor cells and the formation of new blood vessels, a metastasis neoplasm could successfully migrate to the distant organs. As our knowledge, separation from the original tumor, cell adhesion and degradation of the basement membrane were the initial steps of tumor cell metastasis. Cell adhesion to the basement membrane and destroying of basement membrane were the key to tumor metastasis procession. In tumors with high potential invasion and metastasis, the cell adhesion of homogeneity usually declined, whereas the cell adhesion of heterogeneity increased [40,41]. In this study, the reduction of proteoglycans could inhibit the adhesion, invasion and metastasis ability of SACC-M-WJ4 cells, the lung metastasis rate was decreased to 40%, much lower than that of SACC-M cells (100%). In our transient transfection of SACC-83 cells with shRNA-WJ4, the proliferation of tumor cell was inhibited and the cell metastasis ability in vitro was suppressed significantly compared with that of the control (data not shown). The results suggested that proteoglycans perhaps provide an important support in the metastasis of SACC cells.

Although the amount of different proteoglycans secreted by the cells was not tested in this research, the results in previous reports had shown that the main proteoglycans secreted by SACC-M cells were HSPG, few CSPG, biglycan, lumican, dermatan sulfate proteoglycans (DSPG), and some core protein as well [28,39,42]. The proteoglycans reduction was possible the suppression of HSPG to a great degree, which was related closely to the ability of cell migration and invasion. So, SACC-M-WJ4 cells showed obviously lower lung metastasis than that of SACC-M cells in this study.

It has been proved that there were many cell adhesion factors such as growth factors, cytokines and their receptor that located in the GAGs chains. Proteoglycans had a regulatory role of cell adhesion and cell activity [43,44]. It is known that the signal transfer of proteoglycans may mediate the tumor metastasis, whereas HSPG is nearly the most powerful proteoglycans, which carries out this function. It has been known that HSPG synthesized inside the cells stay on the cell membrane for a very short time, and then deposit in the out space of cells. During this process over dozens of HSPG play different functions to regulate the cell metastasis [44-47].

It had been found that over-expression of HSPG promoted the growth of SACC. Over-expression of HSPG is often a prerequisite for the invasion and metastasis of tumor cells. HSPG is not only acted as an important component of basement membrane material, but also involved in the degradation of basement membrane [48,49]. As the most important transmembrane proteoglycans, HSPG plays a decisive role in regulating cell adhesion and transmembrane signal transfer.

Meanwhile, many studies have confirmed that proteoglycans could combine with lots of cell factors such as HGF, bFGF, VEGF, etc. to participate in the formation of induced angiogenesis, which is the necessary process to trigger tumor invasion and migration [50,51]. In the research of myoepithelial salivary tumors, it had been found that the proportion of HSPG/CSPG in the tumor cells was closely related to the tumor metastasis. The up-regulation of HSPG/CSPG proportion enhanced the ability of tumor metastasis, whereas the decline of CSPG/DSPG inhibited tumor metastasis [52,53].

Some scholars had proposed that the proteoglycans secreted by SACC cells provided sufficient nutrition for the proliferation of the tumor cells [54]. Simultaneously the signal receptors and cell adhesion factors holding by proteoglycans could mediate the metastasis of tumor cells and promote the invasion of SACC to distant organs. Some researches also suggested that proteoglycans secreted by SACC cells support the perineural invasion during which energy was poorly supplied by tumor cell [55,56]. In our study, the data implied that the abnormal biosynthesis of proteoglycans in SACC-M cells might be a promoter for the invasion and metastasis of tumor cells.

The reduction of proteoglycans in this study resulted in the inhibition of SACC-M cells adhesion, invasion and migration. The probable reason might be as follows: 1) Cells adhesion factors involved in cell invasion and migration were lost or suppressed by the poor proteoglycans in tumor cells. 2) The decline of proteoglycans on the surface of cytoplasmic membrane or in extracellular matrix inhibited the signal transfer of cells and decreased the cells metastasis. 3) The cell transfer factors could not carry out their function actively without some kind of proteoglycans and their corresponding receptors. It had been known that the abnormal secretion of proteoglycans could result in the dramatically proliferate of cells and this is probably the mechanism of tumorigenesis [44,56]. We also found in other part of our research that the down-regulated proteoglycans had suppressed the proliferation of SACC-M cells dramatically (data not shown). With the decline of proteoglycans, the growth of tumor cells might be suppressed and this might result in the pronounced inhibition of the metastatic activity of SACC cells.

## Conclusions

To Sum up, in this research the knockdown of XYLT-I gene in SACC-M cells by RNAi was effective. The significant inhibition of proteoglycans biosynthesized by SACC-M cells was detected. Our data clearly demonstrated that the reduction of proteoglycans could suppress the biological activities of SACC-M cells including cell adhesion, invasion and lung metastasis. This research not only approved a close relationship between proteoglycans and the metastasis of SACC, but also provided a new idea for the clinical treatment of SACC.

## Competing interests

The authors declare that they have no competing interests.

## Authors' contributions

HS carried out the cell and mice experiments, participated in the construction of shRNA and drafted the manuscript. XW participated in SACC cell culture and the statistical analysis. HL and YH participated in the injections in mice and histological analysis. JW conceived the study, and participated in all the experiment and revised the manuscript. FD provided general support. All authors read and approved the final manuscript.

## Pre-publication history

The pre-publication history for this paper can be accessed here:

http://www.biomedcentral.com/1471-2407/9/456/prepub
